# Synthesis of Defective MOF-801 via Air–Liquid Segmented Flow for Catalytic Transfer Hydrogenation of Furfural

**DOI:** 10.3390/molecules30132697

**Published:** 2025-06-22

**Authors:** Yuxuan Liu, Qiuju Fu, Weijing Niu, Yingxin Zhang, Wenpeng Xie, Huimin Jiang, Liting Yan, Guangda Li, Xuebo Zhao

**Affiliations:** 1 Advanced Materials Institute, School of Materials Science and Engineering, Qilu University of Technology (Shandong Academy of Sciences), Jinan 250353, China; 10431231187@stu.qlu.edu.cn (Y.L.); b20030011@s.upc.edu.cn (W.N.); l2918343623@163.com (Y.Z.); xiewp815@163.com (W.X.); yanlt@qlu.edu.cn (L.Y.); 2 Shandong Provincial Key Laboratory of Chemical Energy Storage and Novel Cell Technology, Qilu University of Technology (Shandong Academy of Sciences), Jinan 250353, China; 3 College of Materials and Engineering, Qingdao University of Science and Technology, Qingdao 266042, China; bh208@qust.edu.cn

**Keywords:** air–liquid segmented flow, furfural, defective MOF-801, catalytic transfer hydrogenation, furfuryl alcohol

## Abstract

As one of the most important platform chemicals, furfural (FAL) can be converted into high-value-added products such as furfuryl alcohol (FOL) through multiple pathways. Zirconium-based MOF-801 demonstrates exceptional catalytic potential for FAL conversion via catalytic transfer hydrogenation (CTH), owing to its unique crystal defects generated during growth. In this study, a series of defective MOF-801 samples were efficiently synthesized using an air–liquid segmented microfluidic technique. The characterization results reveal that the air–liquid segmented flow method not only regulates the defect content of MOF-801 to expose more active sites but also adjusts the crystal size and pore structures by precisely controlling the reaction time. The enhanced defects in MOF-801 significantly improved its catalytic performance. A-MOF-801-64 exhibited the highest activity, achieving over 99% FAL conversion and 98% FOL selectivity under mild conditions (130 °C, 12 h) using isopropanol as the hydrogen donor; this performance surpassed that of other reported Zr-based catalysts. This study will facilitate the practical applications of defect-engineered MOF-801 in upgrading biomass-derived chemicals.

## 1. Introduction

As nature’s most abundant renewable carbon resource, biomass offers cost-effective and ecologically benign advantages over its petroleum-derived counterparts, making it a promising organic carbon platform for sustainable industrial applications [1,2,3]. Furfural (FAL), known as a biorefinery platform compound, is particularly noteworthy due to its lignocellulosic feedstock accessibility and immense potential in yielding high-value chemicals and fuels. As one of the most important platform chemicals [4,5,6], FAL can be converted through various pathways into multiple high-value products, including furfuryl alcohol (FOL), 2-methylfuran (2-MF), levulinic acid (LA), and more [7,8,9]. Of the derivatives mentioned above, FOL holds exceptional industrial significance as a key bio-based furan compound. Its widespread applications, such as plastics, synthetic fibers, liquid fuels, resins, and lubricants, have established its preeminence as the most representative high-value derivative in the furanics value chain [10].

Traditionally, the catalytic hydrogenation of FAL to FOL has predominantly employed molecular hydrogen (H_2_) as the hydrogen donor, typically utilizing noble metal catalysts (e.g., Pd, Pt, and Ru) or non-precious metal catalysts (e.g., Cu, Ni, and Co) [11,12,13,14,15,16]. Although significant progress has been achieved, persistent challenges, including the difficulty in H_2_ transportation, high storage costs, and inherent safety risks in conventional high-pressure hydrogenation processes, have driven the pursuit of developing more efficient and safer alternative strategies. In recent years, catalytic transfer hydrogenation (CTH) employing liquid organic hydrogen donors as alternatives to molecular H_2_ has emerged as a promising research direction. Notably, the Meerwein–Ponndorf–Verley (MPV) reduction—a specific route employing isopropanol as the hydrogen donor—has attracted increasing attention, becoming particularly prized for its outstanding selectivity toward C=O bond hydrogenation in carbonyl compounds. Additionally, the MPV reaction features advantages including mild reaction conditions, avoidance of high-pressure requirements, and enhanced safety in reagent storage and transportation [17,18,19]. A wide variety of catalysts can be employed in MPV reactions, including metals, zeolites, metal alkoxides, metal oxides, and metal–organic frameworks (MOFs) [20,21,22]. Among these, zirconium-based catalysts—encompassing zirconium oxide (ZrO_2_) [23], zirconium hydroxide [24,25], and Zr-based MOFs [26,27]—have undergone rigorous investigation, emerging as predominant research targets in contemporary heterogeneous catalysis studies.

Metal–organic frameworks (MOFs), a rapidly growing class of hybrid porous crystals built from metal nodes and organic linkers, have emerged as versatile functional materials. Owing to their unique properties, such as ultrahigh porosity, tunable pore size, exceptional specific surface area, remarkable thermal/chemical stability, and abundant exposed active sites, MOFs exhibit tremendous potential in catalytic applications [28,29,30,31]. Among them, Zr-MOFs are particularly noteworthy, as they are constructed from Zr_6_(μ-O)_4_(COO)_n_ clusters (where *n* = 6, 8, 10, or 12) coordinated with various carboxylate linkers, which endow them with outstanding stability. During practical MOF crystallization processes, the emergence of structural imperfections and connectivity defects is thermodynamically inevitable. These defect sites are predominantly saturated by hydroxyl groups or coordinated water molecules, which intriguingly endow the materials with unanticipated catalytic functionalities and tunable acid–base characteristics. Therefore, rational defect engineering by introducing structural imperfections has emerged as a crucial strategy for tailoring MOF functionalities, enabling atomic-level control over the physicochemical characteristics. By rationally introducing defects into MOFs, researchers can not only optimize intrinsic properties such as catalysis activity and adsorption capacity but also potentially endow the materials with new functionalities for targeted applications, especially in catalysis [32,33,34,35]. MOF-801, a representative Zr-MOF employing fumarate ions as linkers, has demonstrated wide applicability in gas adsorption, water harvesting, and battery technologies due to its robust thermal and chemical stability [36,37,38,39]. Existing studies have demonstrated that by generating controlled defects in MOF-801, its performance can be significantly enhanced. The exposed open Zr sites can be used for efficiently converting FAL to FOL. Many reports have highlighted that defective MOF-801 holds tremendous research potential [40,41]. However, the controllable preparation of defective MOF-801 remains a significant challenge.

The air–liquid segmented microfluidic method, as an emerging synthetic route, has clear advantages in the preparation of MOFs, including enhanced heat and mass transfer efficiency, precisely controlled reaction conditions, and high product yields [42,43]. In this study, we utilized an air–liquid segmented microfluidic method to prepare a series of defective MOF-801 samples by changing the reactive time (denoted as A-MOF-801-t, where “A” indicates air, and “t” represents reaction time). The obtained defective MOF-801 materials were employed to assess FAL CTH performance using short-chain alcohol as hydrogen donors. Characterizations revealed that compared to solvothermally synthesized MOF-801 (denoted as ST-MOF-801, where ST stands for the solvothermal method), defective A-MOF-801 exhibited higher FOL yields in the FAL CTH reaction. As expected, due to the higher mesoporosity and defect concentration exposing more active sites, FAL molecules can be more readily adsorbed on the catalyst surface, which accounts for significantly enhanced catalytic efficiency.

## 2. Results and Discussion

### 2.1. Characterization of the Catalyst

**Figure 1:**
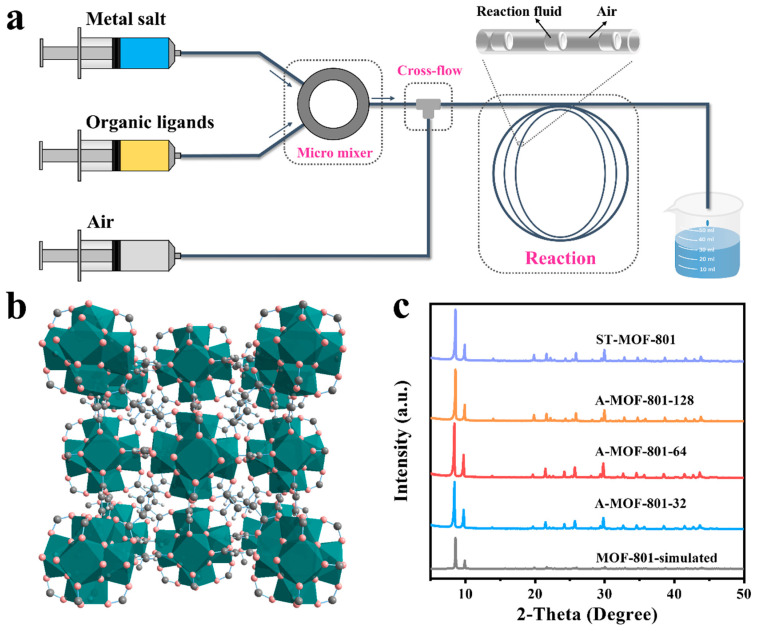
(**a**) Schematic illustration of the air–liquid segmented microfluidic flow system. (**b**) Packing mode presentation of the structure of MOF-801. (**c**) XRD patterns of A-MOF-801-32, A-MOF-801-64, A-MOF-801-128, ST-MOF-801, and the simulated XRD pattern from the crystallographic data of MOF-801.

As shown in Figure 1a, A-MOF-801 samples were synthesized using our laboratory-designed microfluidic flow system. The theoretical pore structures of MOF-801 consist of triangular pores with Zr_6_O_4_(OH)_4_ interconnected with fumarate (with a cubic lattice constant of 17.9090 Å), with microporous diameters of 5.8 Å (Figure 1b and Figure S2). A series of A-MOF-801 samples with different residence times were synthesized by varying the reactant flow rates. Powder X-ray diffraction (PXRD) confirmed the high purity and crystallinity of both A-MOF-801-t and ST-MOF-801 samples (Figure 1c). The diffraction patterns demonstrate excellent agreement between the observed peak positions and those simulated from the MOF-801 crystal structure, with corresponding peak intensities showing close matching. Notably, despite the significantly shorter synthesis time, A-MOF-801 exhibited comparable peak intensities to the ST-MOF-801. The enhanced crystallization efficiency originates from the superior mass and heat transfer properties of the air–liquid segmented flow method, which outperforms conventional solvothermal synthesis, clearly demonstrating the advantages of this approach.

**Figure 2:**
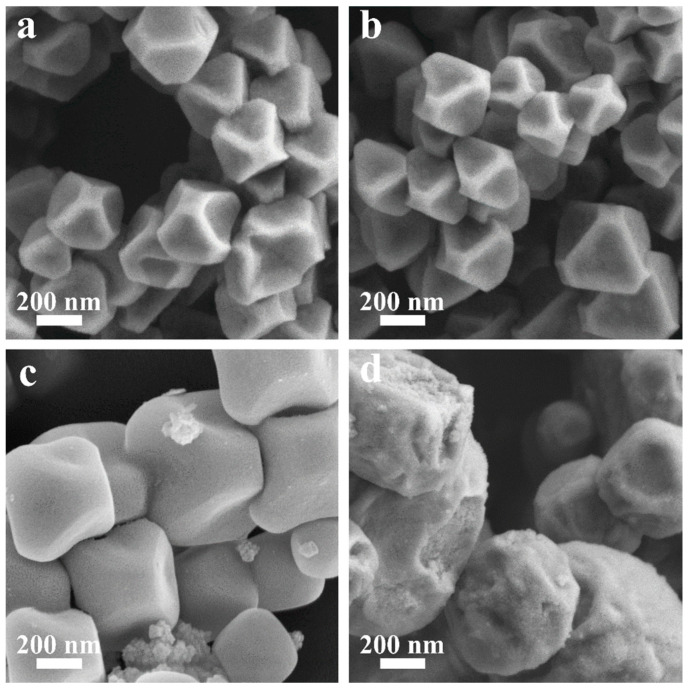
SEM photographs of (**a**) A-MOF-801-32, (**b**) A-MOF-801-64, (**c**) A-MOF-801-128, and (**d**) ST-MOF-801.

The morphology of the obtained MOF-801 products was investigated by scanning electron microscopy (SEM) to further evaluate their quality. As shown in Figure 2a–c, all A-MOF-801 samples exhibited well-defined polyhedral crystals with a uniform particle size distribution and no detectable impurity phases. When the reaction time was 32 min, the crystal showed a uniformly truncated octahedral morphology, showing particles averaging 333 nm in size (Figures S4a and S5a). Extending the reaction time to 64 min, the crystal size gradually increased to about 353 nm (Figures S4b and S5b). As the reaction time increases, the crystals progressively grow. Further prolonging the reaction time to 128 min, the crystal size grew to around 610 nm, with the crystals displaying smooth surfaces and demonstrating well-developed, near-perfect crystalline morphology (Figures S4c and S5c). This growth behavior shows remarkable similarity to the observed trend for polyhedral HKUST-1 crystals synthesized via our group’s previous microdroplet flow method [44]. Notably, ST-MOF-801 exhibited broadly similar morphology to A-MOF-801 samples (Figure 2d). However, ST-MOF-801 demonstrated significantly rougher crystal surfaces, a non-uniform particle size distribution, and irregular crystal shapes. Furthermore, the particles showed poor dispersibility with frequent occurrences of particle aggregation. In contrast to A-MOF-801, ST-MOF-801 exhibited larger particle sizes of approximately 860 nm with rough surfaces, irregular morphologies, and numerous surface damages (Figures S4d and S5d). These results further validate the advantages of modulating MOF morphology and the particle size of the air–liquid segmented flow synthesis method. Within the air–liquid segmented flow system, the reaction solution forms discrete liquid segments under shear forces, which effectively minimizes axial dispersion. Additionally, the internal recirculation patterns within each segment enhance radial mixing. These conditions enable the air–liquid segmented flow method to achieve superior mass transfer efficiency compared to the traditional solvothermal method, thereby enabling the controllable evolution of both the morphology and size distribution of the resultant materials.

**Figure 3:**
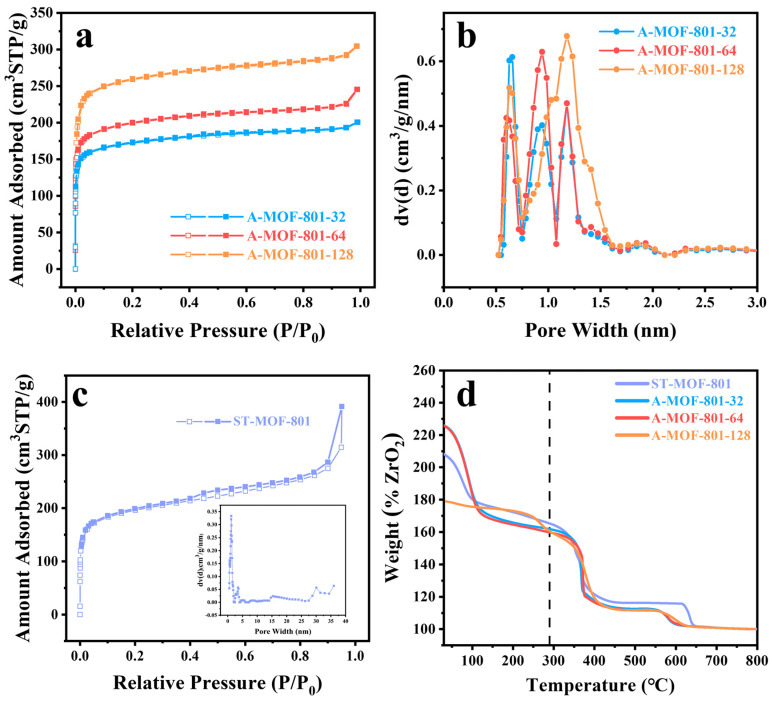
(**a**) N_2_ adsorption isotherms at 77 K for A-MOF-801-t; (**b**) Pore size distribution profiles of A-MOF-801-t; (**c**) N_2_ adsorption isotherm at 77 K for ST-MOF-801 with inset showing its pore size distribution, and (**d**) TGA curves under air atmosphere for ST-MOF-801 and A-MOF-801-t.

To investigate the pore structure evolution of A-MOF-801-t samples with different reaction times, N_2_ adsorption measurements at 77 K were conducted. All synthesized samples displayed characteristic Type I adsorption isotherms, as evidenced in Figure 3a,c. The isotherms demonstrate a sharp uptake at low relative pressures, confirming the existence of micropores, followed by a plateau region at intermediate pressures. Notably, a slight upward deviation appears at high relative pressures due to capillary condensation in the mesopores formed between crystalline particles, reaffirming that both A-MOF-801-t and ST-MOF-801 are characterized by the narrow pore size distribution of microporous materials. The pore size analysis clearly demonstrates that the pore diameters of A-MOF-801-t samples display three peaks with different ratios between 0.5 and 2.0 nm (Figure 3b), which are significantly larger than the pore size of the original MOF-801 [36]. This distinct difference confirms that the air–liquid segmented flow method effectively modulates the pore structure of MOF-801 crystals. The observed pore enlargement can be attributed to the unique crystallization environment created by the air–liquid segmented flow method. Compared to the conventional solvothermal method, the extremely short reaction time of the air–liquid segmented flow system prevents sufficient time for crystal defect repair and maturation. Meanwhile, the continuous and uniform movement of the liquid segments maintains a stable crystal growth environment, resulting in more homogeneous pore size distribution throughout the crystal. The mesoporosity of A-MOF-801 increases when the reaction time is extended from 32 min to 64 min but shows a decrease upon further prolongation to 128 min (Figure S4, Table S1). This trend indicates that the crystal growth of A-MOF-801 essentially completes at 64 min. The next stage is the self-repairing of the crystal, where partial mesopores within the A-MOF-801 crystals disappear due to defect repair and structural perfection. In comparison with the A-MOF-801 samples, ST-MOF-801 exhibits significantly higher mesoporosity and contains a considerable proportion of large mesopores exceeding 15 nm (Figure 3c). From the point of view of catalytic applications, larger pore sizes are generally more favorable for catalytic reactions since many reactants are size-restricted from accessing the internal pores of microporous materials. However, the presence of excessively large mesopores can paradoxically reduce catalytic activity by diminishing the specific surface area of the catalyst. The appropriate pore structure of A-MOF-801 facilitates reactant diffusion to internal active sites, thereby improving the catalytic performance of the FAL hydrogenation reactions.

To systematically characterize the defect content in A-MOF-801-t samples, thermogravimetric analysis (TGA) was performed. As shown in Figure 3d, each sample exhibited a significant weight loss at around 100 °C, corresponding to the evaporation of surface-adsorbed water molecules. A minor weight loss observed near 150 °C corresponds to the removal of residual solvent molecules. Differential thermal analysis revealed the framework collapse temperatures for ST-MOF-801, A-MOF-801-32, A-MOF-801-64, and A-MOF-801-128 were 362 °C, 367 °C, 366 °C, and 376 °C, respectively (Figure S5). The decomposition temperature of the prepared MOF-801 is almost the same as the value reported in the literature, confirming its excellent thermal stability despite the changing pore size distribution of A-MOF-801-t. The defect rate of MOFs can be estimated from the weight loss of their TGA curves [26]. Through normalization calculations of the MOFs’ weight loss (corresponding to ligand decomposition) and residual weight (primarily ZrO_2_) on the TG curve, the relative content of ligands to metals in the MOFs can be determined, thereby obtaining the W_MOF_/W_ZrO₂_ ratio. Compared to the theoretical W_MOF_/W_ZrO₂_ ratio of MOF-801 (180%), the value in A-MOF-801-t samples is significantly lower, indicating the presence of ligand defects [45,46,47]. The ligand deficiency rate of A-MOF-801-t was calculated based on the measured W_MOF_/W_ZrO₂_ ratios, where the defect concentration of A-MOF-801-64 was the highest among the samples (Table S2). In addition, as the synthesis duration extended beyond 32 min to 64 min, the ligand deficiency rate showed an increasing trend, which differs from conventional crystallization processes. This phenomenon can be primarily attributed to our method of controlling reaction time by adjusting the flow rate—longer reaction times correspond to a slower movement of the liquid segments. During the reaction, gravity causes the solid products generated to concentrate mainly in the lower part of the liquid segments, which disrupts the flow balance of the liquid segments. Consequently, the probability of ligand mismatch or deficiency increases, leading to higher ligand deficiency rates in the final product. Ligand deficiency induces the formation of mesopores within the microporous framework, and the higher the ligand defects, the more mesopores the sample contains.

**Figure 4:**
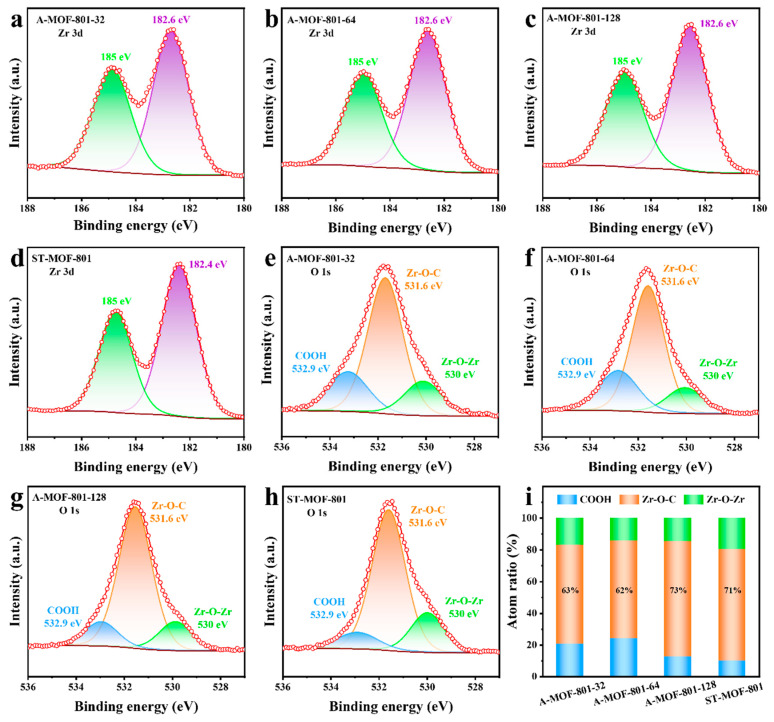
High-resolution Zr 3d XPS spectra of (**a**) A-MOF-801-32; (**b**) A-MOF-801-64; (**c**) A-MOF-801-128, and (**d**) ST-MOF-801. High-resolution O 1s XPS spectra of (**e**) A-MOF-801-32; (**f**) A-MOF-801-64; (**g**) A-MOF-801-128, and (**h**) ST-MOF-801. (**i**) The contents of three types of oxygen groups (COOH, Zr-O-C, and Zr-O-Zr refer to the coordinated-free carboxylate groups, the coordinated carboxylate groups, and the bridged group on SBUs, respectively).

X-ray photoelectron spectroscopy (XPS) was conducted on all synthesized MOF-801 samples to investigate their chemical composition. The chemical species present at the C 1s, Zr 3d, and O 1s peak positions were identified and annotated (Figure 4 and Figure S8). All peaks were corrected for C-C peak positions (284.8 eV) (Figure S9). The spectral results confirm that the chemical states and elemental valences of the A-MOFs synthesized via the air–liquid segmented flow method remained unchanged compared to ST-MOF-801. In the high-resolution Zr 3d XPS spectra (Figure 4a–d), all samples exhibited similar peak distributions, with only minor binding energy shifts. The two characteristic peaks centered at 182.6 eV and 185.0 eV were assigned to the 3d_5/2_ and 3d_3/2_ of Zr-O clusters, respectively, consistent with previously reported Zr-MOFs [39]. Unlike the Zr 3d spectra, the high-resolution O 1s XPS spectra show remarkable differences among the synthesized MOF-801 products (Figure 4e–h). Three characteristic peaks appeared at binding energies of 532.9 eV, 531.6 eV, and 530.0 eV, corresponding to C-O, C=O, and Zr-O bonds, respectively. These peaks were assigned to coordinated-free carboxylate groups (COOH), coordinated carboxylate groups (Zr-O-C), and bridging groups (Zr-O-Zr) in the framework of MOF-801 (Figure S10). Notably, the relative peak area of Zr-O-C initially decreased and then increased as the reaction time of A-MOFs was extended, while the peak intensity of Zr-O-Zr showed an inverse trend (Figure 4i). In Zr-MOFs, Zr-O-C groups represent the connections between metal centers and organic linkers. Therefore, the relative content of Zr-O-C can serve as an indicator of the degree of crystal defects. The lower the Zr-O-C relative content, the higher the defects in the sample. Among the synthesized samples, A-MOF-801-64 has the lowest Zr-O-C content and therefore the highest defect content, which is consistent with the TGA results.

### 2.2. Evaluation of the Catalytic Performance of A-MOF-801-t

**Scheme 1:**
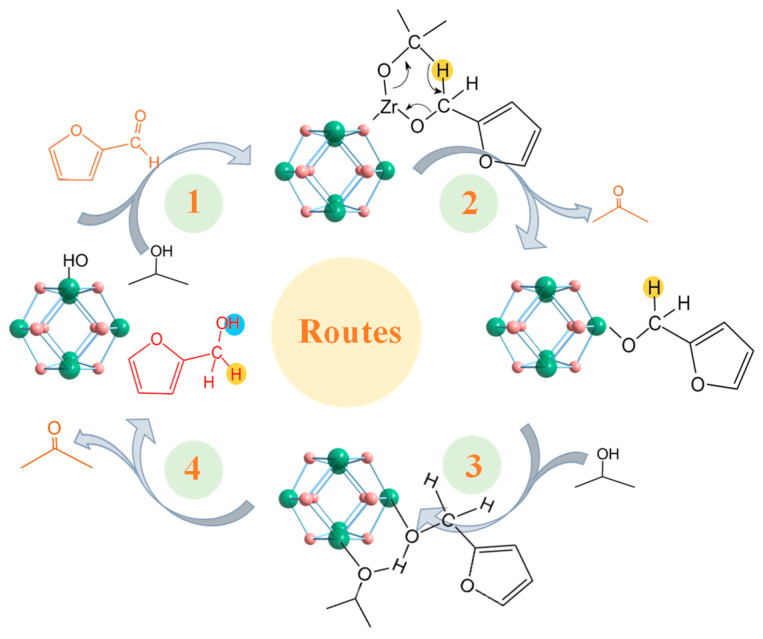
Proposed mechanism for converting FAL into FOL catalyzed by open Zr metal sites via CTH reaction with 2-propanol as the hydrogen donor.

**Figure 5:**
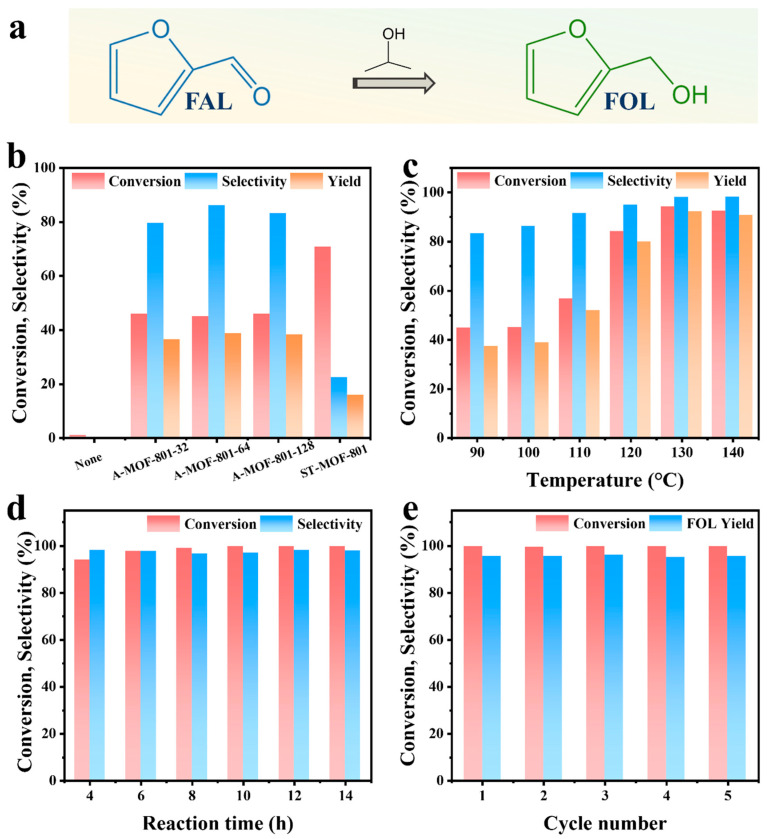
(**a**) FAL reaction routes on MOF-801. (**b**) Catalytic performance of ST-MOF-801 and A-MOF-801-t (reaction condition: 0.1 g furfural, 0.1 g catalyst, 50 mL isopropanol, 160 °C, 4 h). (**c**) Effect of reaction temperature (reaction time: 4 h). (**d**) Effect of reaction time (reaction temperature: 130 °C). (**e**) Cycling stability of A-MOF-801-64: 130 °C, 12 h.

We evaluated the catalytic performance of the synthesized A-MOF-801-t samples using isopropanol as a hydrogen donor. Using isopropanol as the hydrogen donor as well as reaction solvent, FAL can easily be converted into FOL (Figure 5a). Drawing upon prior experimental findings and literature reports [48,49,50], we gained fundamental insights into the CTH process of FAL on MOF-801. MOF-801 comprises octahedral Zr_6_O_4_(OH)_4_ clusters bridged by twelve carboxylate linkers. The high coordination number of the Zr-O clusters leads to the rare exposure of open Zr sites for catalysis. Therefore, defect engineering is necessary for obtaining MOF-801 with open Zr sites. Generally speaking, the open Zr sites obtained from defects are occupied by hydroxyl groups in water molecules, forming a Zr-OH structure. The catalytic pathway initiates with the adsorption of both isopropanol and FAL on the catalyst surface of Zr-OH groups (Scheme 1, Route1). This adsorption process occurs primarily at the Lewis acidic sites on Zr-O clusters within MOF-801, which are exposed due to ligand deficiency. Subsequently, the Zr sites form metal alkoxides with the hydroxyl groups of isopropanol. Concurrently, the carbonyl oxygen of FAL becomes activated and stabilized through interaction with these Lewis acidic Zr clusters. Both reactants are anchored at the catalytic sites to establish a six-membered ring transition state, enabling hydrogen transfer from the isopropanol-derived alkoxide to the FAL carbonyl group. Next, acetone was removed and an intermediate was formed (Scheme 1, Route 2). Subsequently, another isopropanol participated to obtain a six-membered ring transition state (Scheme 1, Route 3). Following the hydrogen transfer process, FOL and acetone desorb from the catalyst surface, thereby regenerating the active Zr sites in MOF-801 for subsequent catalytic cycles (Scheme 1, Route 4). In the absence of a catalyst, no FOL products are generated, which confirms the catalytic dependence of the FAL CTH reaction. From the catalytic performance of the prepared MOF-801 samples, the conversion of ST-MOF-801 was higher, but its selectivity was significantly lower than that of the A-MOF-801 (Figure 5b). This difference in performance is because the Zr site in ST-MOF-801 is not sufficiently exposed. However, ST-MOF-801 exhibits a specific surface area comparable to A-MOF-801-64; the active sites required for the selective hydrogenation of FAL to FOL remain scarce. Notably, the catalytic performance of A-MOF-801 varies with different reaction times. Among these, A-MOF-801-64 demonstrated optimal catalytic activity, exhibiting both the highest FOL selectivity (86.2%) and yield (38.72%). In contrast, the relatively low selectivity of A-MOF-801-32 may be attributed to fewer exposed active sites resulting from its lower specific surface area. With a high specific surface area, small particle size, and abundantly exposed defect sites, A-MOF-801-64 has the highest catalytic activity. Therefore, A-MOF-801-64 emerges as the optimal catalyst for the present reaction system.

### 2.3. Influence of Reaction Conditions

The influence of reaction temperature on A-MOF-801-64’s catalytic performance was systematically investigated. As illustrated in Figure 5b, the reaction temperature exerted a pronounced influence on the catalyst’s performance toward FAL conversion within the 110–120 °C range. Under a mild reaction condition of 100 °C, FAL conversion reached only 44.92%. A pronounced enhancement in catalytic activity was observed when the temperature reached 120 °C, with a dramatic conversion increase to 84.15%. The conversion reached a maximum value of 94.13% at 130 °C. Beyond 130 °C, further elevated temperatures resulted in a decrease in conversion, which was attributed to the occurrence of side reactions under higher temperatures. Moreover, the FOL selectivity reached its maximum above 130 °C, exhibiting no significant variation with additional temperature increases. Also, the reaction yield reached its maximum (92.33%) at 130 °C. Therefore, 130 °C was identified as the optimal reaction temperature for A-MOF-801-64 in this reaction.

A-MOF-801-64’s catalytic activity versus reaction time appears in Figure 5d. The results reveal that the FAL conversion initially showed relatively fast growth; then, the growth rate began to slow down after 8 h, reaching a plateau at 10 h (>99%). In contrast, the selectivity of FOL first decreased and then rebounded, reaching a peak value of 98.09% after 12 h, which exhibited better performance than other Zr-based catalysts reported in previous studies (Table S3). However, upon extending the reaction time to 14 h, the FOL selectivity decreased again, indicating increased byproduct formation with prolonged reaction time. These results established 12 h as the optimal reaction duration for further investigations. Accordingly, the optimal reaction conditions of A-MOF-801-64 for converting FAL to FOL were defined as 130 °C and 12 h.

Catalyst stability is an important indicator for industrial applications. Catalyst stability was assessed for A-MOF-801-64 under the established optimal conditions (Figure 5e). The catalyst was separated by centrifugation, washed three times with methanol, and dried at 80 °C until the next use. After five consecutive catalytic cycles, the conversion of A-MOF-801-64 was maintained at about 95%, and the FOL selectivity was stable, demonstrating no significant loss of activity. Meanwhile, a series of characterizations including PXRD, SEM, and XPS were performed on the cycled A-MOF-801-64 catalyst (Figures S11–S13). The PXRD analysis revealed the complete retention of the original framework’s characteristic diffraction peaks, with no phase impurities or peak broadening detected, confirming excellent crystallinity preservation. SEM imaging demonstrated identical morphological features before and after cycling, showing no signs of structural degradation. XPS spectra exhibited virtually unchanged binding energies and elemental distributions, further verifying the material’s chemical stability under cycling conditions. Collectively, these results provide compelling evidence for the exceptional cycling stability of A-MOF-801-64.

## 3. Experimental Section

### 3.1. Synthesis Methodology

#### 3.1.1. Synthesis of A-MOF-801-t

The A-MOF-801 samples were synthesized using a microfluidic flow system. In a typical synthesis, 1.78 g of ZrOCl_2_·8H_2_O and 0.64 g of fumaric acid were separately dissolved in 11 mL of N, N-dimethylformamide (DMF) and 4 mL of formic acid, respectively, to form a uniform precursor solution. The solutions were then pumped into the pre-mixing section, where the two solutions were uniformly mixed. Subsequently, air was introduced into the tubing, and the reaction liquid formed uniformly stable small liquid segments 2 mm in length under the shear force of the gas (Figure S1). When these segments entered the heating zone, a crystallization reaction occurred rapidly due to the excellent heat/mass transfer efficiency. The reaction time was determined by the controllable injection rate, with the reaction temperature set at 120 °C and a constant air-to-liquid injection velocity ratio of 3:1. After the reaction, the product was isolated via centrifugation, filtered, and washed using methanol as the eluent in Soxhlet extraction. The obtained product was vacuum-dried at 80 °C for 12 h, yielding a white powder.

#### 3.1.2. Synthesis of ST-MOF-801

A solution containing metal salt (1.78 g of ZrOCl_2_·8H_2_O) and an organic ligand (0.64 g of fumaric acid) was thoroughly mixed by stirring before being transferred into a Teflon-lined autoclave. The mixture was reacted at 120 °C for 24 h. After cooling to ambient temperature, the product was isolated via centrifugation and vacuum-dried for 12 h, yielding white powder.

### 3.2. Structural Characterization

Powder X-ray diffraction (PXRD) analyses were performed using an X-ray diffractometer (X’ pert Pro, PANalytical, Almelo, The Netherlands) to determine the crystallinity and phase purity of the samples. Using a Cu Kα radiation source (λ = 0.15406 nm) operated at 40 kV and 30 mA, diffraction data were collected from 5° to 50°. The resulting patterns were then compared with the simulated spectrum of MOF-801. The sample morphology and particle size distribution were characterized by field-emission scanning electron microscopy (SEM, S-4800, Hitachi, Tokyo, Japan) at an acceleration voltage of 20 kV. Nitrogen adsorption–desorption measurements were performed at 77 K using a low-temperature physisorption analyzer (Quantachrome, Boynton Beach, FL, USA). The material’s specific surface area, pore size distribution, and pore structure were examined using non-local density functional theory (NLDFT) and Brunauer–Emmett–Teller (BET) methods. Thermogravimetric analysis (TGA) was carried out using a NETZSCH TG 209F3 analyzer under an air atmosphere, with a heating rate of 5 °C min^−1^ from room temperature to 800 °C. The measurements were conducted to determine the decomposition/desorption temperature ranges of guest species or other unstable components in the structure, as well as the structural collapse temperature. X-ray photoelectron spectroscopy (XPS) measurements were performed using an EscaLab 250Xi photoelectron spectrometer. The C 1s peak at 284.6 eV was used as an internal reference to calibrate the binding energy scale to compensate for surface charge-induced shifts.

### 3.3. Catalytic Activity Tests

FAL transfer hydrogenation tests were performed in a mechanically stirred stainless steel autoclave (E50, Senlang, Beijing, China). For the experiment, a 1:1 mass ratio of catalyst to FAL, along with 50 mL of isopropanol, was charged into the reactor. After sealing the autoclave, we maintained the hydrogenation reaction at the target temperature, continuously stirring at 800 rpm. Upon reaching the reaction time, 0.3 mL of the reaction mixture was collected, subjected to a 100 nm syringe-driven filter to obtain a clear solution, and analyzed via gas chromatography (GC) (Fuli, Taizhou, China) equipped with a flame ionization detector (FID) and a capillary column. Qualitative analysis of the products was accomplished by comparing their retention times with those of reference standards, while quantitative analysis was based on the respective signal response values. All the experimental results were obtained from single experimental runs, except for the cycling tests. The GC operating conditions were as follows: a helium carrier gas, a capillary column temperature of 90 °C, an FID temperature of 250 °C, and an injector temperature of 200 °C. The conversion rates of FAL (C_FAL_) and product selectivity (S_products_) were calculated with the following formulae:

(1) $$C_{FAL}=\frac{n_{FAL,0}-n_{FAL,t}}{n_{FAL,0}}\times 100\%$$

(2) $$S_{products}=\frac{n_{products}}{n_{FAL,0}-n_{FAL,t}}\times 100\%$$

In which n_FRR,0_ represents the initial molar quantity of FAL before the reaction, n_FAL,t_ denotes the molar quantity of FAL during the reaction, and S_products_ indicates the molar quantities of individual reaction products.

## 4. Conclusions

In summary, we presented a novel strategy for synthesizing a series of defective A-MOF-801 samples using the air–liquid segmented flow method. This strategy effectively regulated the particle size and defect concentration of the MOF-801 by altering the reaction time. The prepared defective A-MOF-801 samples were used for the CTH reaction to efficiently convert FAL to FOL. The results show that the A-MOF-801 samples prepared under different reaction times exhibited different defect concentrations and pore structures, which were closely related to the catalytic performance. Notably, the A-MOF-801-64 sample obtained exhibited the highest catalytic performance regarding FAL to FOL conversion, achieving more than 99% FAL conversion and 98% FOL selectivity under mild conditions (130 °C for 12 h) using 2-propanol as the hydrogen donor. The abundance of Zr active sites due to ligand defects endows A-MOF-801-64 with excellent catalytic activity. Meanwhile, the superior catalytic activity of A-MOF-801-64 is closely related to its reduced particle size, high specific surface area, and abundant micro–meso pore structure, which facilitates the exposure of catalytic sites and the contact between FAL and active sites. The air–liquid segmented strategy for the acquisition of defective MOF-801 demonstrates cost-effectiveness, rapidness, and high efficiency, enabling the possibility of designing MOF defect engineering and providing valuable guidance for the synthesis and application of other defective MOFs with significant catalytic potential.

## Data Availability

Data will be made available on request.

## Supplementary Materials

The following supporting information can be downloaded at: https://www.mdpi.com/article/10.3390/molecules30132697/s1, Figure S1: The liquid segments in the air–liquid segmented microfluidic system during operation: (a) before entering the oven, (b) after entering the oven; Figure S2: The crystal structure of MOF-801 on the (1 1 1) crystal plane; Figure S3: Metric of the unit cell and table of atomic coordinates of the structural model of Zr-fum MOF as obtained by structural modelling; Figure S4: Statistical analysis of the size of A-MOF-801-t with residence time of (a) 32 min; (b) 64 min; (c) 128 min; (d) ST-MOF-801; Figure S5: SEM images for particle size statistics: (a) A-MOF-801-32; (b) A-MOF-801-64; (c) A-MOF-801-128; (d) ST-MOF-801; Figure S6: Statistical analysis of BET surface area (left) and mesopore size distribution (right) of A-MOF-801-t; Figure S7: The diagram on stable presence temperature of ST-MOF-801 and A-MOF-801-t frame structures; Figure S8: XPS survey spectrum of ST-MOF-801 and A-MOF-801-t samples; Figure S9: The narrow scan in C 1s of MOF-801 samples; Figure S10: Schematic representation of the proposed surface chemical species of C 1s, O 1s, and Zr 3d peaks for the synthesized materials; Figure S11: XRD patterns of A-MOF-801-64 before and after five consecutive catalytic cycles; Figure S12: SEM photographs of A-MOF-801-64 after five consecutive catalytic cycles; Figure S13: XPS survey spectrum of A-MOF-801-64 before and after five consecutive catalytic cycles; Table S1: The pore structure parameters of A-MOF-801-t and ST-MOF-801; Table S2: The ligand content and ligand defect rate of A-MOF-801-t series products and ST-MOF-801 calculated from TGA curves; Table S3: The particle size statistics of A-MOF-801-32. Table S4: The particle size statistics of A-MOF-801-64. Table S5: The particle size statistics of A-MOF-801-32. Table S6: The particle size statistics of A-MOF-801-64. Table S7: CTH of FFR by various Zr-based catalysts. References [36,49,51,52,53,54,55,56] are cited in the supplementary materials.
