# Supplementary material for: Synthesis of Defective MOF-801 via Air–Liquid Segmented Flow for Catalytic Transfer Hydrogenation of Furfural
Source: Molecules. 2025 Jun 22;30(13):2697. doi: 10.3390/molecules30132697 (PMC12251738; doi:10.3390/molecules30132697)
Supplement: Supplementary file 1 [file molecules-30-02697-s001.zip › molecules-3681943-supplementary.pdf]

Supporting Information

for

Synthesis of Defective MOF-801 via Air–liquid Segmented Flow for Catalytic Transfer Hydrogenation of Furfural

Contents:

1. Supplementary Experimental Section

2. Supplementary Figures

3. Supplementary Tables

1. Supplementary Experimental Section

1.1 Synthesis of A-MOF-801-t

Experiments were performed using a self-assembled microdroplet reaction device consisting of three syringe pumps, polytetrafluoroethylene (PTFE) reaction tubing, a premixer, heating zone, and product collection zone. $ZrOCl_2 \cdot 8H_2O$ (1.78 g, 5.5 mmol) and fumaric acid (0.64 g, 5.5 mmol) were separately dissolved in DMF (11 mL) and formic acid (4 mL) to form homogeneous solutions. The mixed solution entered the reaction tubing through a 1 mm inner diameter stainless steel tube and met the air at a T-junction, maintaining a constant air-to-liquid velocity ratio of 3:1. Under air shear force, the solution was segmented into uniform liquid segments. These segments continuously flowed through 2 mm inner diameter/3 mm outer diameter PTFE tubing placed in a 120 °C oven. After reaction in the tubing to form white products, the material exited the heating zone. The air was vented and products were collected in centrifuge tubes. The collected products were cooled to room temperature, centrifuged, and then dried under vacuum at 80 °C for 12 hours to obtain white powder.

1.2 Synthesis of ST-MOF-801

$ZrOCl_2 \cdot 8H_2O$ (1.78 g, 5.5 mmol) and fumaric acid (0.64 g, 5.5 mmol) were separately dissolved in DMF (11 mL) and formic acid (4 mL) to form homogeneous solutions. The solutions were stirred for 30 min and then moved to a Teflon-lined reactor. Subsequently, the reactor was programmed at 120°C for 24 h. The collected products were cooled to room temperature, centrifuged, and then dried under vacuum at 80 °C for 12 hours to obtain white powder.

2. Supplementary Figures

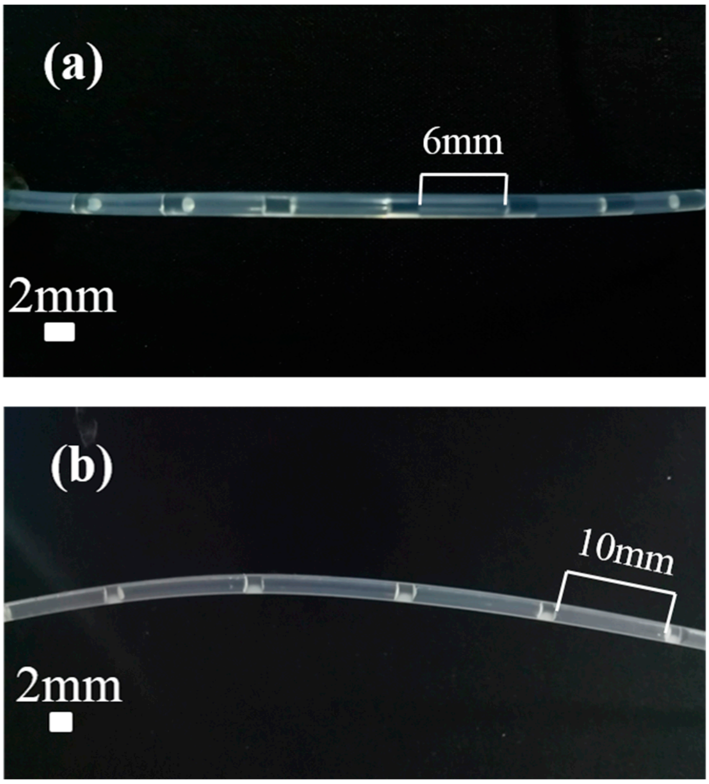

Fig. S1 The liquid segments in the air-liquid segmented microfluidic system during operation: (a) before entering the oven, (b) after entering the oven.

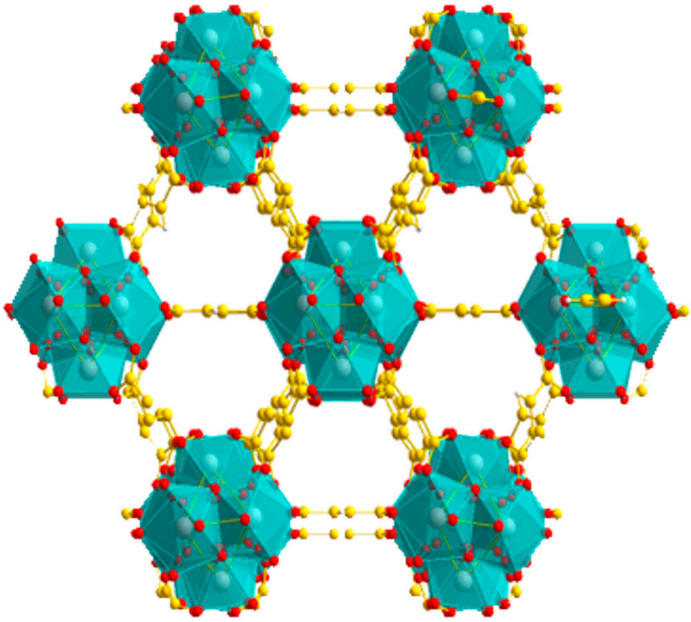

Fig. S2 The crystal structure of MOF-801 on the (1 1 1) crystal plane.

Cubic crystal system, space group *P*-3*n* (No. 201)
a = *b* = c = 17.909 Å, *V* = 5744 Å^3
$\alpha = \beta = \gamma = 90.0$ °

symbol	element	x / *a*	y / *b*	z / *c*
O1	O	0.66425	0.27385	0.9548
O2	O	0.87028	0.25685	0.93686
O3	O	0.77331	0.36957	0.9371
O4	O	0.75915	0.16374	0.95501
O5	O	0.83158	0.31899	0.80795
C6	C	0.59165	0.27636	0.93835
C7	C	0.93878	0.24856	0.90848
Zr8	Zr	0.76152	0.26135	0.88368
C9	C	0.53831	0.28103	0.00421
C10	C	0.00446	0.24819	0.96178
O11	O	0.81986	0.18014	0.81986

Fig. S3 Metric of the unit cell and table of atomic coordinates of the structural model of Zr-fum MOF as obtained by structural modelling [36].

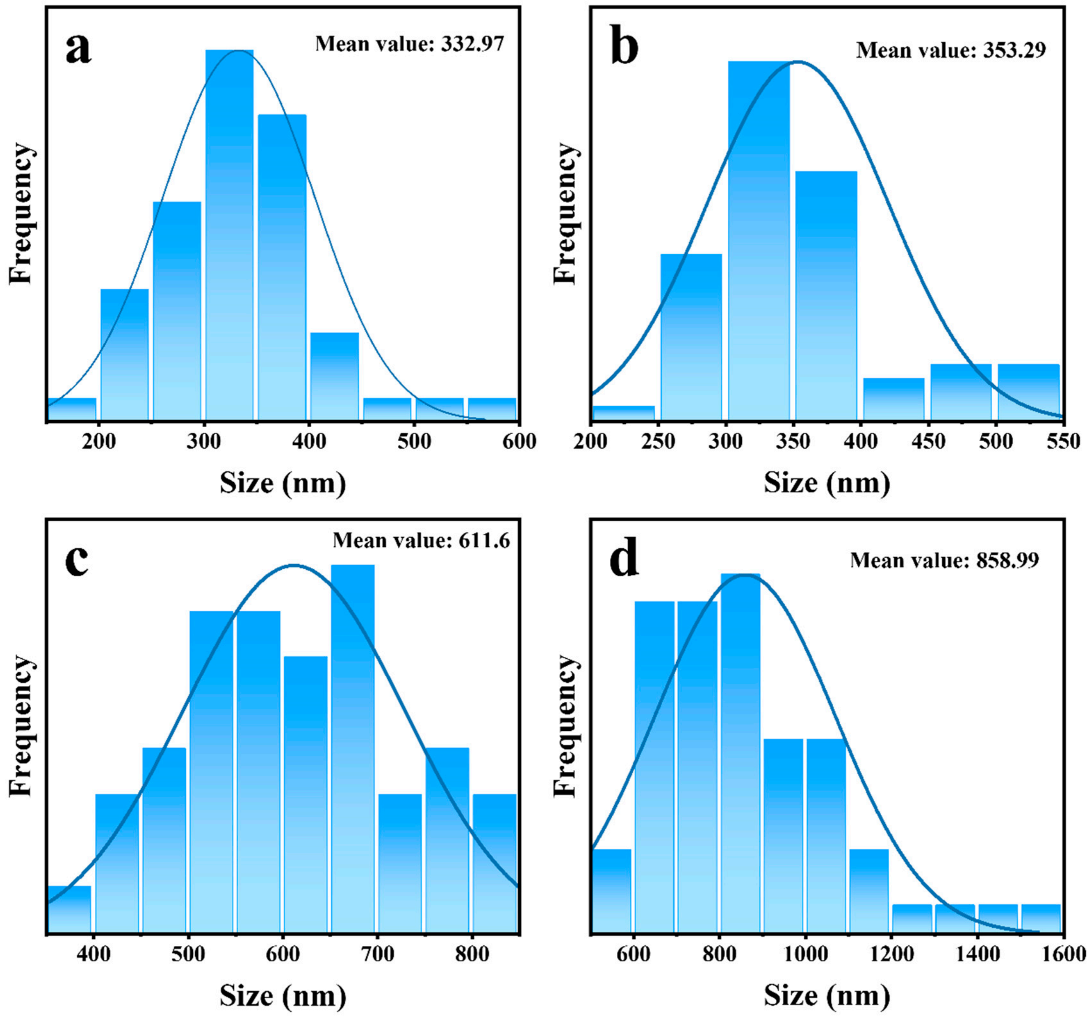

Fig. S4 Statistical analysis of the size of A-MOF-801-t with residence time of (a) 32 min; (b) 64 min; (c) 128 min; (d) ST-MOF-801.

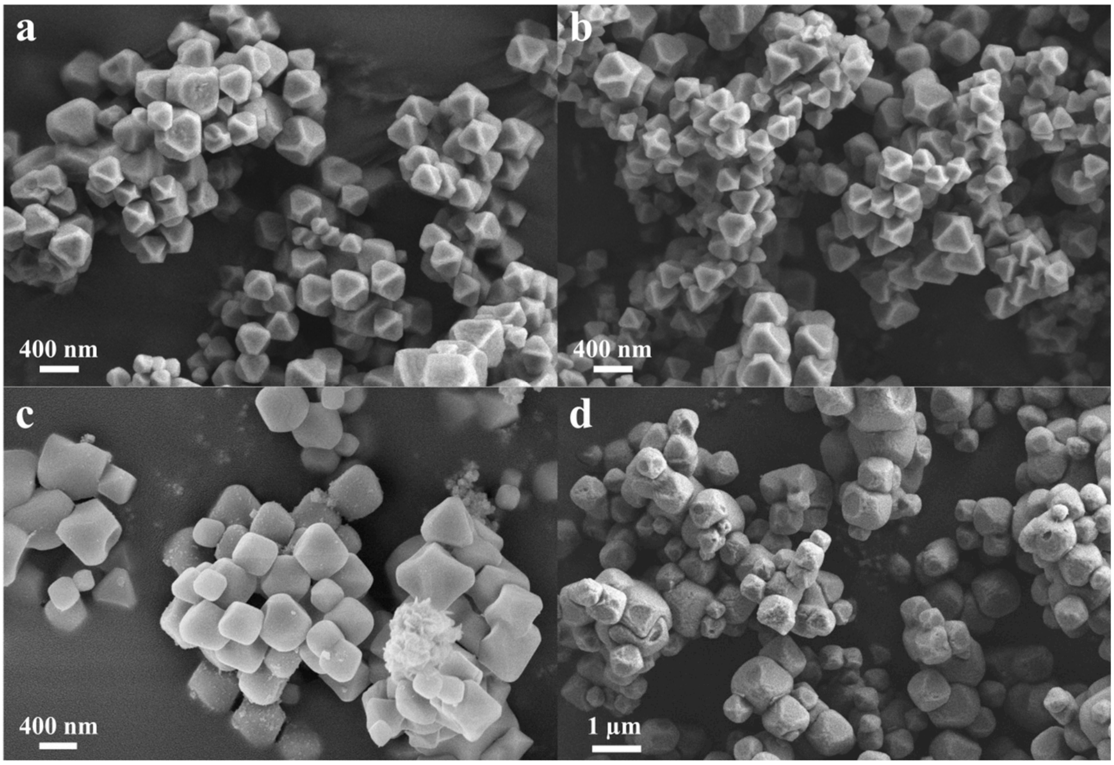

Fig. S5 SEM images for particle size statistics: (a) A-MOF-801-32; (b) A-MOF-801-64; (c) A-MOF-801-128; (d) ST-MOF-801.

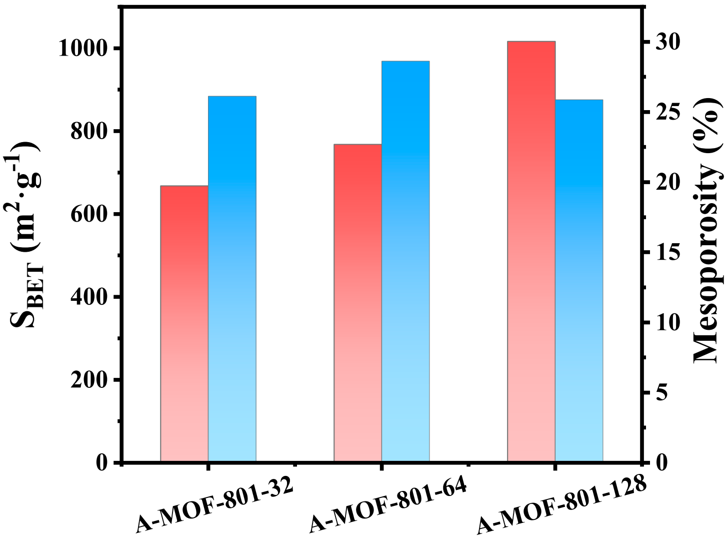

Fig. S6 Statistical analysis of BET surface area (left) and mesopore size distribution (right) of A-MOF-801-t.

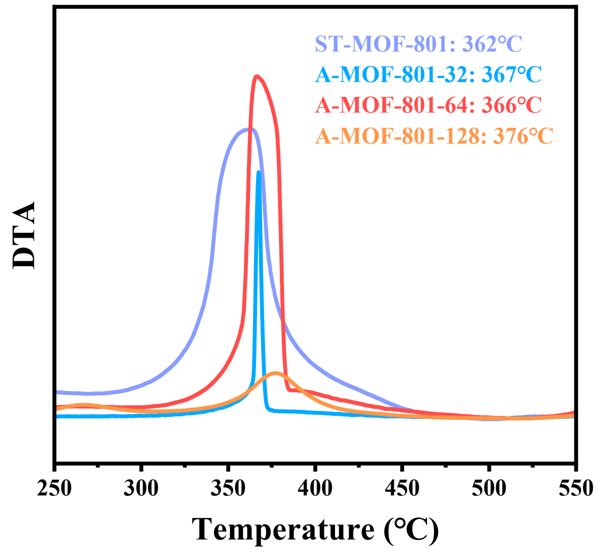

Fig. S7 The diagram on stable presence temperature of ST-MOF-801 and A-MOF-801-t frame structures.

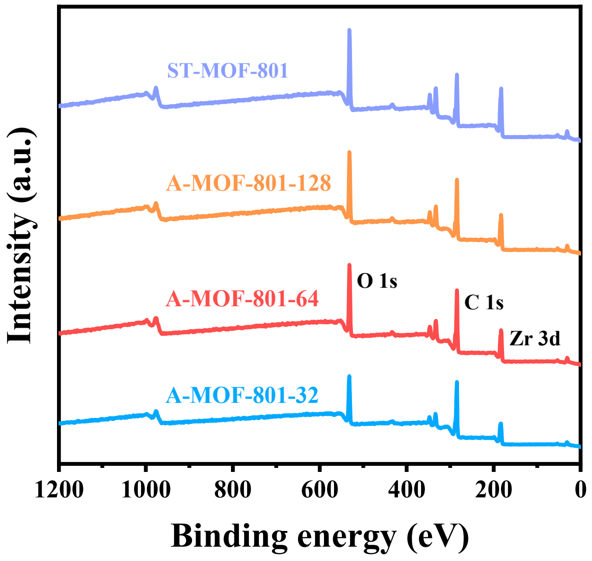

Fig. S8 XPS survey spectrum of ST-MOF-801 and A-MOF-801-t samples.

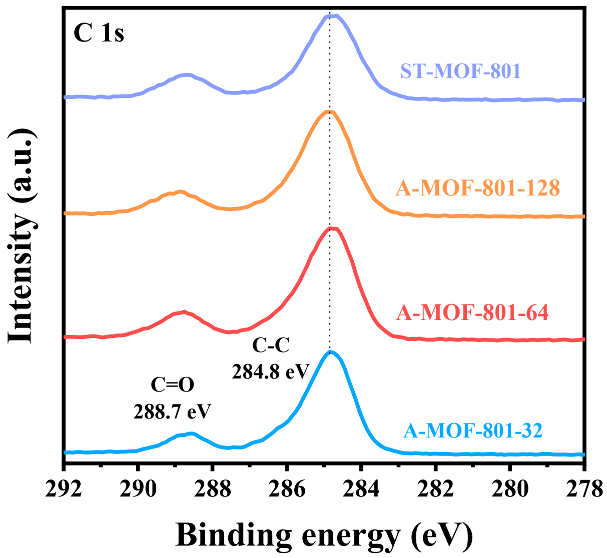

Fig. S9 The narrow scan in C 1s of MOF-801 samples.

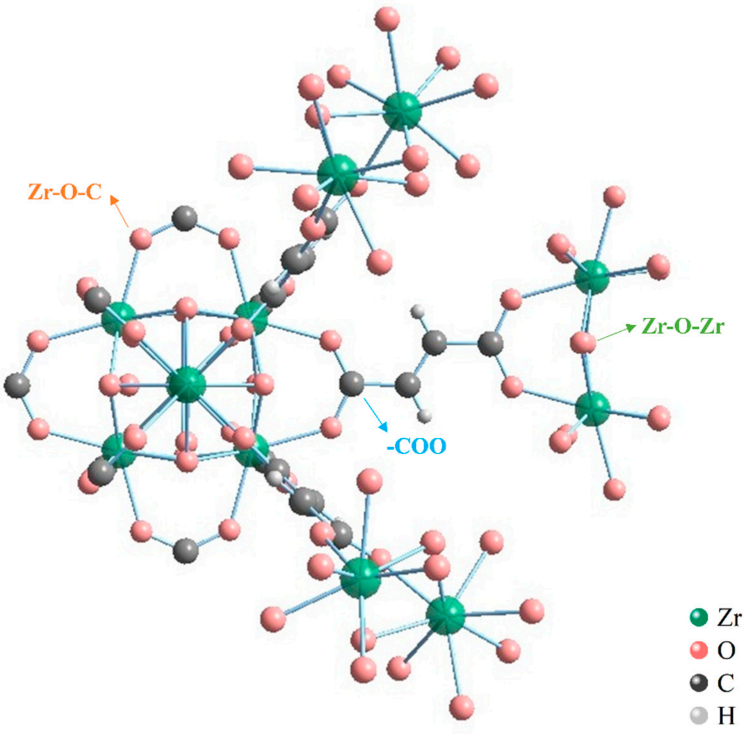

Fig. S10 Schematic representation of the proposed surface chemical species of C 1s, O 1s, and Zr 3d peaks for the synthesized materials.

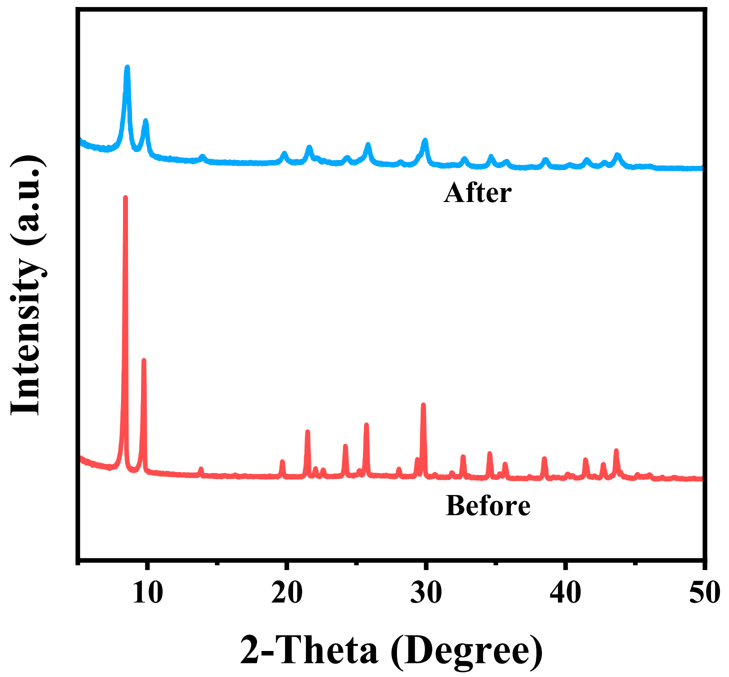

Fig. S11 XRD patterns of A-MOF-801-64 before and after five consecutive catalytic cycles.

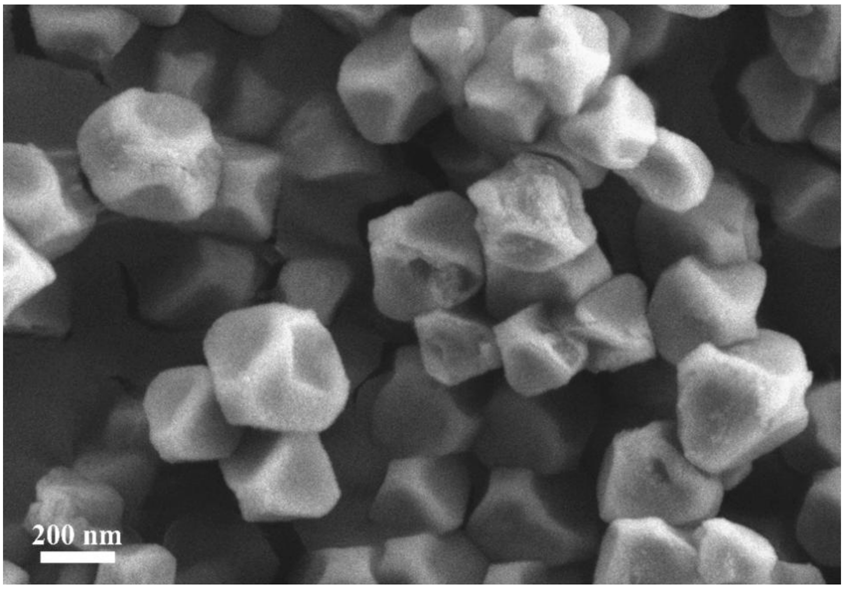

Fig. S12 SEM photographs of A-MOF-801-64 after five consecutive catalytic cycles.

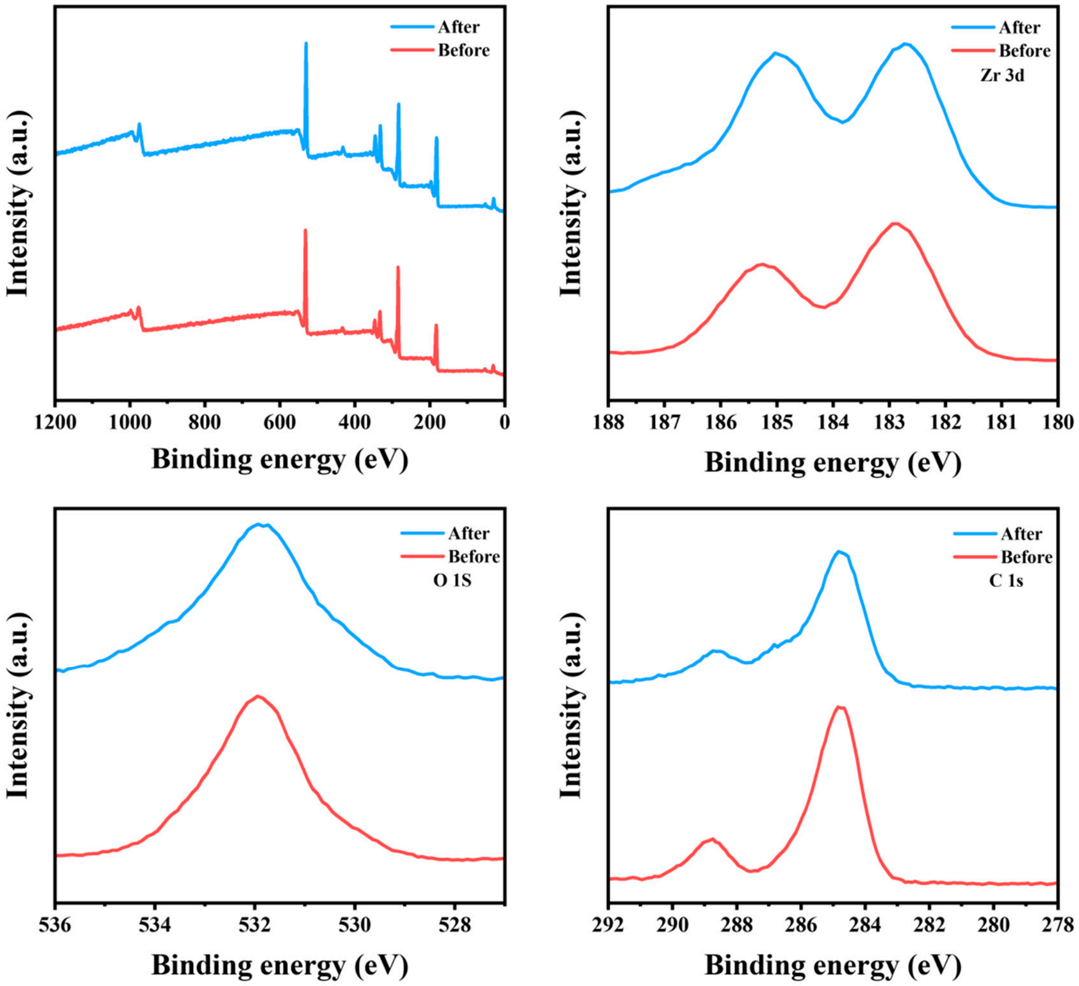

Fig. S13 XPS survey spectrum of A-MOF-801-64 before and after five consecutive catalytic cycles.

3. Supplementary Tables

Table S1. The pore structure parameters of A-MOF-801-t and ST-MOF-801.

MOFs	Residence time (min)	S_{BET} ($m^2 \cdot g^{-1}$)	V_{total} ($cm^3 \cdot g^{-1}$)	Mesoporosity (%)
A-MOF-801-32	32	667.92	0.3100	26.13
A-MOF-801-64	64	767.71	0.3797	28.63
A-MOF-801-128	128	1016.61	0.4708	25.87
ST-MOF-801	1440	728.04	0.877	75.03

Table S2. The ligand content and ligand defect rate of A-MOF-801-t series products and ST-MOF-801 calculated from TGA curves.

MOFs	W_{MOF}/ W_{ZrO_2}	Loss of ligand content	Ligand defect rate
A-MOF-801-32	161.74	61.74	18.26
A-MOF-801-64	159.72	59.72	20.28
A-MOF-801-128	160.26	60.26	19.74
ST-MOF-801	165.25	65.25	14.75

The initial ligand content is uniform by weight at 290 °C.

Table S3. The particle size statistics of A-MOF-801-32.

No.	Size (nm)	No.	Size (nm)	No.	Size (nm)
1	382.8	20	397.61	39	283.14
2	320.62	21	356.38	40	288.37
3	361.97	22	399.59	41	242.26
4	564.31	23	452.89	42	338.85
5	332.58	24	403.63	43	249.03
6	530.07	25	408.01	44	295.3
7	395.84	26	348.32	45	283.12
8	326.5	27	365.47	46	311.12
9	361.74	28	319.85	47	279.43
10	202.32	29	349.88	48	335.6
11	212.83	30	298.7	49	338.85
12	263.53	31	286.4	50	280.41
13	371.73	32	212.4	51	356.16
14	415.83	33	196.71	52	221.47
15	376.85	34	417.52	53	319.85
16	303.71	35	353.19	54	282.6
17	308.26	36	376.48	55	378.92
18	305.75	37	312.91		
19	314.55	38	321.22		

Table S4. The particle size statistics of A-MOF-801-64.

No.	Size (nm)	No.	Size (nm)	No.	Size (nm)
1	410.15	24	339.46	47	301.19
2	311.56	25	348.46	48	317.54
3	283.25	26	496.84	49	360.8
4	281.94	27	407.86	50	388.41
5	322.84	28	323.59	51	377.01
6	286.21	29	312.13	52	394.06
7	301.31	30	355.92	53	281.63
8	379.98	31	337.62	54	303.66
9	280.14	32	364.92	55	289.6
10	360.97	33	369.78	56	314.33
11	284.76	34	342.44	57	301.78
12	266.81	35	506.47	58	385.3
13	334.45	36	318.23	59	381.88
14	331.73	37	377.79	60	336.13
15	328.64	38	284.89	61	360.95
16	332.7	39	237.9	62	371.04
17	255.97	40	544.3	63	453.42
18	502.5	41	348.25	64	312.16
19	294.43	42	317.81	65	367.54
20	352.24	43	494.12	66	323
21	382.93	44	439.97	67	389.73
22	342.93	45	526.19	68	291.23
23	334.69	46	463.05		

Table S5. The particle size statistics of A-MOF-801-32.

No.	Size (nm)	No.	Size (nm)	No.	Size (nm)
1	748.28	17	513.29	33	525.33
2	786.21	18	774.73	34	605.79
3	667.77	19	529.19	35	651.97
4	817.88	20	652.45	36	567.71
5	816.67	21	739.49	37	568.06
6	693.41	22	428.19	38	444.11
7	731.13	23	677.44	39	602.2
8	604.43	24	466.58	40	526.09
9	664.09	25	482.14	41	525.02
10	505.64	26	592.94	42	595.46
11	598.3	27	501.52	43	496.9
12	787.22	28	778.85	44	646.96
13	694.8	29	554.59	45	622.52
14	804.11	30	611.82	46	555.1
15	692.04	31	356.36		
16	446.27	32	482.57		

Table S6. The particle size statistics of A-MOF-801-64.

No.	Size (nm)	No.	Size (nm)	No.	Size (nm)
1	853.93	22	1430.32	43	752.2
2	745.3	23	1061.65	44	945.62
3	741.25	24	844.96	45	706.1
4	592.66	25	871.92	46	1505.05
5	531.74	26	1052.95	47	743.61
6	769.28	27	923.9	48	617.19
7	694.61	28	731.87	49	606.39
8	687.23	29	779.21	50	665.91
9	817.25	30	766.66	51	1113.47
10	878.65	31	798.63	52	867.81
11	1289.08	32	840.71	53	896.28
12	655.93	33	914.58	54	997.86
13	1006.17	34	982.79	55	1307.62
14	1000.56	35	1020.19	56	878.91
15	831.28	36	696.86	57	704.16
16	658.94	37	1010.7	58	912.16
17	948.63	38	1110.5	59	634.91
18	877.1	39	1192.97	60	636.49
19	644.21	40	505.6	61	846.83
20	1056.5	41	604.42		
21	756.87	42	881.22		

Table S7. CTH of FFR by various Zr-based catalysts.

Catalysts	T (°C)	t (h)	Con. (%)	Sele. (%)	Ref.
Zr@Co-2	160	4	93.9	97.3	[49]
Zr-20@PAN	150	2	98.4	91.8	[51]
3ZSB	160	3	100	97	[52]
ZrNPs-lignin/PAN	150	3	97.1	98.8	[53]
Zr-FDCA	150	3	100	100	[54]
ZrO_2	180	0.17	94	55	[55]
Zr_5Al_5	130	3	96	92.7	[23]
UIO-$S_{0.6}$	150	6	100	93.6	[56]
A-MOF-801-64	130	12	>99	98	This work

Reference

36. Wissmann, G.; Schaate, A.; Lilienthal, S.; Bremer, I.; Schneider, A. M.; Behrens, P., Modulated synthesis of Zr-fumarate MOF. *Microporous and Mesoporous Materials* **2012,** 152, 64-70.
49. Hou, P.; Ma, M. W.; Zhang, P.; Cao, J. J.; Liu, H.; Xu, X. L.; Yue, H. J.; Tian, G.; Feng, S. H., Catalytic transfer hydrogenation of furfural to furfuryl alcohol using easy-to-separate core-shell magnetic zirconium hydroxide. *New Journal of Chemistry* **2021,** 45, (5), 2715-2722.
51. Lin, W. S.; Cheng, Y.; Liu, H.; Zhang, J. H.; Peng, L. C., Catalytic transfer hydrogenation of biomass-derived furfural into furfuryl alcohol over zirconium doped nanofiber. *Fuel* **2023,** 331.
52. Kumaravel, S.; Alagarasan, J. K.; Yadav, A. K.; Ali, W.; Lee, M. Y.; Khan, M. E.; Ali, S. K.; Bashiri, A. H.; Zakri, W.; Balu, K., Highly selective catalytic transfer hydrogenation of biomass derived furfural to furfural alcohol over Zr/SBA-15 catalysts. *Journal of Physics and Chemistry of Solids* **2024,** 186.
53. Lin, W. S.; Wang, Y.; Zhang, J. H.; Liu, H.; Peng, L. C., Lignin-assembled zirconium-based PNA nanofiber for the catalytic transfer hydrogenation of furfural into furfuryl alcohol. *Sustainable Energy & Fuels* **2023,** 7, (15), 3716-3726.
54. Cheng, Y.; Liu, Y.; Zhang, J.; Huang, R.; Wang, Y.; Cao, S.; He, L.; Peng, L., Acetic acid-regulated mesoporous zirconium-furandicarboxylate hybrid with high lewis acidity and lewis basicity for efficient conversion of furfural to furfuryl alcohol. *Renewable Energy* **2022,** 184, 115-123.
55. Saotta, A.; Allegri, A.; Liuzzi, F.; Fornasari, G.; Dimitratos, N.; Albonetti, S., Ti/Zr/O Mixed Oxides for the Catalytic Transfer Hydrogenation of Furfural to GVL in a Liquid-Phase Continuous-Flow Reactor. *Chemengineering* **2023,** 7, (2).
23. García-Sancho, C.; Jiménez-Gómez, C. P.; Viar-Antuñano, N.; Cecilia, J. A.; Moreno-Tost, R.; Mérida-Robles, J. M.; Requies, J.; Maireles-Torres, P., Evaluation of the ZrO_2/Al_2O_3 system as catalysts in the catalytic transfer hydrogenation of furfural to obtain furfuryl alcohol. *Applied Catalysis a-General* **2021,** 609.
56. Wu, J.; Liang, D.; Song, X.; Liu, T.; Xu, T.; Wang, S.; Zou, Y., Sulfonic groups functionalized Zr-metal organic framework for highly catalytic transfer

hydrogenation of furfural to furfuryl alcohol. *Journal of Energy Chemistry* **2022,** 71, 411-417.
